# DKVMN&MRI: A new deep knowledge tracing model based on DKVMN incorporating multi-relational information

**DOI:** 10.1371/journal.pone.0312022

**Published:** 2024-10-30

**Authors:** Feng Xu, Kang Chen, Maosheng Zhong, Lei Liu, Huizhu Liu, Xianzeng Luo, Lang Zheng

**Affiliations:** 1 Jiangxi Provincial Education Institute, Jiangxi, China; 2 College of Computer and Information Engineering, Jiangxi Normal University, Jiangxi, China; Jordan University of Science and Technology Faculty of Computer and Information Technology, JORDAN

## Abstract

Knowledge tracing is a technology that models students’ changing knowledge state over learning time based on their historical answer records, thus predicting their learning ability. It is the core module that supports the intelligent education system. To address the problems of sparse input data, lack of interpretability and weak capacity to capture the relationship between exercises in the existing models, this paper build a deep knowledge tracing model DKVMN&MRI based on the Dynamic Key-Value Memory Network (DKVMN) that incorporates multiple relationship information including exercise-knowledge point relations, exercise-exercise relations, and learning-forgetting relations. In the model, firstly, the Q-matrix is utilized to map the link between knowledge points and exercises to the input layer; secondly, improved DKVMN and LSTM are used to model the learning process of learners, then the Ebbinghaus forgetting curve function is introduced to simulate the process of memory forgetting in learners, and finally, the prediction strategies of Item Response Theory (IRT) and attention mechanism are used to combine the similarity relationship between learners’ knowledge state and exercises to calculate the probability that learners would correctly respond during the subsequent time step. Through extensive experiments on three real-world datasets, we demonstrate that DKVMN&MRI has significant improvements in both AUC and ACC metrics contrast with the latest models. Furthermore, the study provides explanations at both the exercise level and learner knowledge state level, demonstrating the interpretability and efficacy of the proposed model.

## Introduction

**Fig 1:**
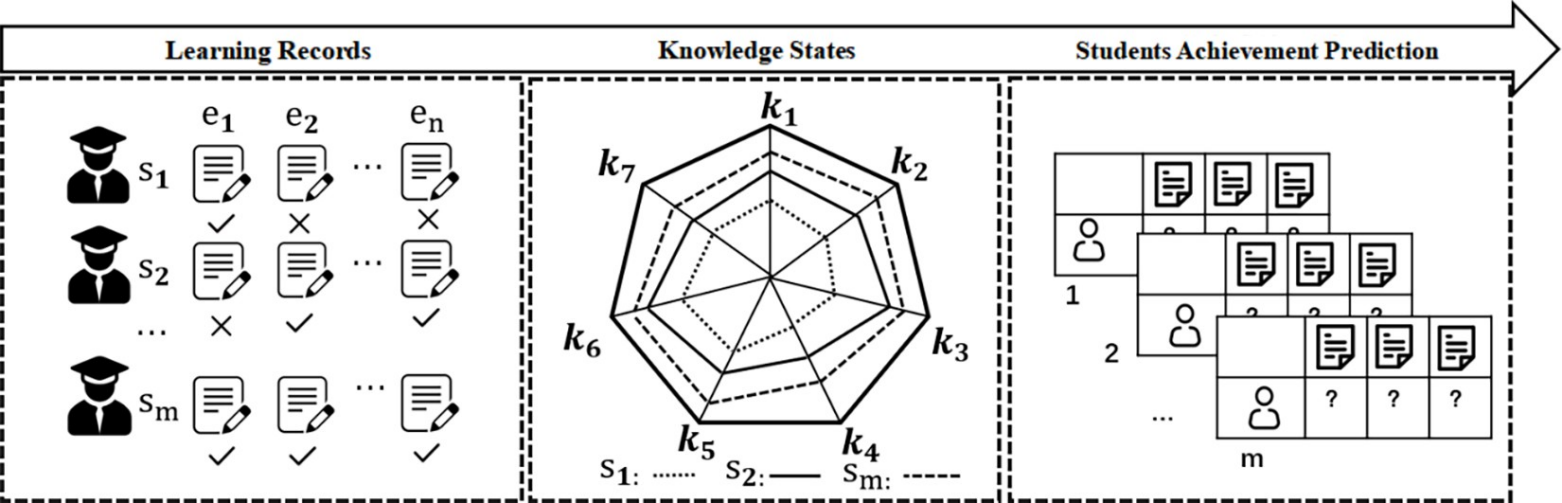
Schematic diagram of knowledge tracing.

With the popularity of online education, the demand for technology to accurately analyze student characteristics and provide personalized education to students is also increasing. Education platform needs to accurately and effectively monitor students’ knowledge state according to their learning trajectories, and then provide personalized teaching services for students. Knowledge tracking is to solve this problem, the core of which is to model students’ knowledge state with learning time based on their historical answer records, so as to predict their answer performance. This provides basic technical support for constructing personalized online education. Specifically, the process of knowledge tracking is shown in Fig 1. Learning records reflect students’ real learning level. Through predefined models, their mastery of knowledge points can be quantified from the learning records, and their performance in unknown exercises can be predicted. This allows for the assessment of students’ current cognitive levels, enabling the learning system to recommend learning resources that match the current level of the students’ knowledge, thereby achieving the goal of personalized learning.

The task of modeling and predicting human learning involves multiple fields, including education, cognitive, neuroscience, and psychology science [1]. Research in education and psychology has revealed that human learning performance is influenced by various factors. For example, in item response theory (IRT) [2], students’ performance prediction is influenced by students’ learning ability and exercises factors (such as difficulty, discrimination, etc.). Furthermore, learning is a complex process involving knowledge consolidation and forgetting, and students’ knowledge levels change continuously over time. Therefore, accurately describing and assessing students’ knowledge states is a complex task.

Currently, research on knowledge tracing is primarily focused on three directions: probabilistic models, logical models, and deep learning-based models. Probabilistic models assume that the learning process follows a Markov process, thus providing statistically interpretable results. On the other hand, logical models constitute a class of models based on logical functions, with the fundamental principle being the use of mathematical functions to represent students’ learning ability parameters and exercise parameters, thereby inferring the probability of providing accurate answers to exercises. In contrast, deep learning-based knowledge tracing models stand out for their powerful feature extraction capabilities, enabling them to capture more complex relationships between knowledge points and exercises, including but not limited to the association between exercises and knowledge points, and the similarity between exercises. Compared to traditional logical and probabilistic models, this method doesn’t need expert annotation of domain knowledge and demonstrates superior predictive performance. Consequently, it has become a hot issue in current knowledge tracing research.

However, deep learning-based knowledge tracing models currently face challenges in interpretability due to their inherent black-box nature. Deep learning models will have very limited practical application if students’ mastery of each knowledge point cannot be precisely measured. Furthermore, existing methods largely focus on the correlations between exercises and knowledge points, while neglecting correlations between exercises, learning and forgetting. The correlations between exercises are an important consideration for accurately predicting students’ future performance. For example, when students successfully answer a question containing a certain knowledge point, they are likely to perform well when facing another question involving the same knowledge point. Simultaneously, modeling the knowledge state of learners is essentially a process of continuous learning and forgetting. Knowledge learned in the past or experiences in solving problems may be forgotten over time, while regular learning and problem-solving may lead to relatively consolidated knowledge states. Therefore, the relationship between learning and forgetting is also a crucial factor that needs to be considered. Additionally, the use of one-hot encoding for model inputs results in excessively sparse data, which is a common issue in current deep learning models.

To solve the above issues, this research suggests a deep knowledge tracing model based on DKVMN that incorporates multi-relational information. Firstly, by introducing the test-knowledge point vector encoded by the Q matrix [3] as the model input, this input method not only alleviates the problem of data sparsity but also takes into account the correlation between exercises and knowledge points. Based on the knowledge points involved, difficulty, and similarity of the exercises, the relationship between exercises is calculated. Then, each knowledge point is mapped into the knowledge space by DKVMN, where each dimensional vector denotes the respective knowledge point’s mastery level. The model updates the level of mastery for the corresponding knowledge according to the student’s performance in answering questions. After that, the student’s knowledge level at each moment is dynamically quantified by a layer of Long Short-Term Memory (LSTM) [4]. The Ebbinghaus forgetting curve function is introduced to portray the effect of forgetting on the learning process. Finally, this paper proposes two prediction strategies based on IRT and attention mechanism, where each parameter of IRT (difficulty, learning ability, etc.) has an interpretable impact on the prediction performance of the model, and the interpretability of the model can be enhanced by combining it with traditional psychometric theory. The attention mechanism-based prediction technique not only considers the impact of current students’ cognitive level on a prediction but also incorporates the answering experience of similar questions in the exercise records. Overall, this paper’s primary contributions consist of the following:

1. Using the Q-matrix not only alleviates the issue of sparse data, but also enables the mining of the mapping relationship between knowledge points and exercises, by mapping the one or more knowledge points contained in exercises to the knowledge space;
2. Introduction of the Ebbinghaus forgetting curve function to model the effect of forgetting on learning, and the past problem-solving experience is exponentially decayed based on the time step length;
3. Incorporating an attention mechanism to capture the similarity between exercises, giving a method to calculate the similarity of exercises based on the difficulty of the exercises and the similarity of knowledge points, which enhances the ability of DKVMN to capture the differences between exercises;
4. Two prediction strategies based on Item Response Theory (IRT) and attention mechanism are proposed: an IRT-based prediction strategy that improves the interpretability of the model; and an attention-based prediction strategy that enables the model to focus on both the current learner’s knowledge state and important past knowledge states;
5. A deep knowledge tracing model based on DKVMN incorporating multi-relationship information (DKVMN&MRI) is constructed, and experiments on several public datasets confirm the model’s superiority.

In this paper, the first section will introduce the work related to knowledge tracing, the second section will define the knowledge tracing problem and the modelling idea of DKVMN&MRI, the third section will elaborate on the implementation steps and prediction process of DKVMN&MRI, the fourth section will give the experimental results and model performance analysis. Finally, the fifth section will give the conclusion and future research work.

## Background

The earliest research on knowledge tracing can be traced back to the 1990s. These studies typically describe the learning process as a hidden Markov model and use the Bayesian formula to calculate learners’ mastery levels of knowledge points. In 1995, Corbett et al. [5] introduced the Bayesian knowledge tracing model into intelligent tutoring systems. Since then, there has been a growing recognition of the importance of knowledge tracing, and increasingly researchers have begun to focus on and delve into this field. In the field of psychometrics, IRT plays a crucial role in educational assessment and measurement. Some researchers have further expanded the parameters based on IRT, for example, Koedinger et al. [6] from Carnegie Mellon University incorporated factors such as learning rate and number of practice attempts into IRT. Pavlik et al. [7], building on the work of Koedinger et al., further subdivided the number of practice attempts into correct attempts and incorrect attempts. These studies, through parameter expansion, have further enhanced the applicability of IRT. However, whether it is the Bayesian knowledge tracing model or the extended models based on IRT, they all need the domain knowledge marked by experts, such as the mapping association between questions and knowledge points.

Researchers have been paying more attention to deep learning in recent years because of its powerful feature extraction capabilities. In 2015, Chris et al. [8] from Stanford University first proposed utilizing Recurrent Neural Networks (RNNs) [9] to model students’ learning processes, achieving significantly superior results compared to classic machine learning techniques. Inspired by Memory-Augmented Neural Networks (MANN) [10], Shi et al. proposed a DKVMN model based on memory networks in 2017. This model introduces an external memory module to store knowledge concepts and update learners’ mastery of corresponding knowledge points, effectively modeling learners’ grasp of each knowledge point. However, this approach overlooks the rich relationships that may exist between exercises, as even exercises with the same knowledge points may contain biases due to differences in difficulty or discrimination. In 2019, Pandey et al. [11] utilized the Transformer model in the field of knowledge tracing. In 2020, Ghosh et al. [12] introduced the Context-aware Attention-based Knowledge Tracing (AKT) model, which effectively addresses the long-term dependency issues of RNNs. In 2022, Lee et al. [13] demonstrated promising performance by integrating attention mechanisms with contrastive learning and adversarial training.

From a data structure perspective, knowledge points can potentially be structured as graphs, and there may be rich dependency relationships between knowledge points. In 2019, Nakagawa et al. [14] proposed Graph-Based Knowledge Tracing (GKT) based on graph neural networks. In GKT, knowledge points and their dependency relationships are modeled as graph nodes and edges, redefining knowledge tracing as a time-series node classification issue in graph neural networks (GNN) [15]. In 2023, Zheng et al. [16] considered the impact of forgetting behavior and temporal characteristics of student response sequences on cognitive states, utilizing graph neural networks for modeling.

In the work considering the differences between exercises, Su et al. [17] proposed an Exercise-Enhanced Recurrent Neural Network (EERNN) in 2018, which effectively exploiting the semantic information of exercises. Firstly, they transformed the M-word sequences of exercise texts into embedded vectors using Word2Vec [18] technology, and then input them into a bidirectional LSTM to obtain the exercises’ final semantic embedding representation. This design helps to avoid biases in predictions caused by significant semantic differences between exercises with the same knowledge points. However, EERNN faces the challenge of explaining the final knowledge state hidden in a vector, which makes it difficult to quantify a student’s mastery of each knowledge point. To address this issue, in 2019, Liu et al. [19] improved upon EERNN and proposed a new model, Exercise-Aware Knowledge Tracing (EKT). The novel aspect of EKT is its ability to accurately track a student’s mastery of every knowledge point by mapping each knowledge point’s state to its associated vector in the knowledge space. Taking into account the fine-grained timing influenced by different cross skills, Wang et al. [20] proposed a Hawkes Knowledge Tracing (HawkesKT) model based on point processes in 2021.

In considering the individual learning abilities of students, Minn et al. [21] proposed a model called Deep Knowledge Tracing with Dynamic Student Classification (DKT-DSC) in 2018, which can assess students’ learning abilities. Through the use of K-means clustering [22], the model assigns students to groups based on similar abilities at each time interval. On the other hand, in 2021, Sun et al. [23] proposed a knowledge tracing algorithm called DKVMN with Learning Ability Integration (DKVMN-LA) based on DKVMN. In 2021, Long et al. [24] introduced individual cognitive modules and exercise-sensitive modules tailored for different students, accompanied by a thorough evaluation. In 2022, Long et al. [25] proposed a collaborative knowledge tracing approach that integrates inter-student and intra-student information.

While deep learning-based techniques have demonstrated better prediction accuracy than conventional probabilistic and logical models, it is challenging to interpret the hidden vectors of the model as knowledge states due to neural networks’ black box character. Therefore, such models also have certain limitations [26].

## Problem definition and main ideas

This section defines the knowledge tracing issue and outlines the key concepts for knowledge tracing modeling in this study.

### Problem definition

One way to approach knowledge tracing is as a guided sequence prediction problem. Assuming a set of students **S** and a set of exercises **E** in a learning system, the learning sequence for a student is represented as **X = {(q**_**1**_**, *k***_**1**_**, r**_**1**_**), (q**_**2**_**, *k***_**2**_**, r**_**2**_),**…,(q**_***t***_, **k**_*t*_, **r**_*t*_**),…,(q**_***N***_, **k**_***N***_, **r**_***N***_**)},** where the tuple (**q**_***t***_, **k**_***t***_**, r**_***t***_**)** represents the learning interaction of the student at time step **t**, where **q**_***t***_ is the exercise completed by the student, **k**_***t***_ is the knowledge point contained in the exercise, and **r**_***t***_ denotes the score obtained by the student for that exercise. **N** represents the length of the learning interaction sequence. Then, students’ knowledge state at every moment is extracted through the preset model, which is usually implicit. By predicting the student’s answer score at the **t+1** moment, this implicit knowledge state can be revealed to achieve the purpose of assessing the student’s cognitive level.

### Main ideas

In response to the issues of input data sparsity, inability to capture the relationships between exercise items, and lack of interpretability in traditional DKVMN, this paper models the learner’s learning process and knowledge tracking based on the following ideas, building upon the foundation of DKVMN.

Firstly, using the Q-matrix, the exercise-knowledge point relationship is mapped into a vector of the same dimension as the quantity of knowledge points, which can incorporate the information of the exercise-knowledge point relationship while alleviating data sparsity; secondly, attention mechanisms are introduced to capture the similarity relationship between exercises based on knowledge points and difficulty aspects; furthermore, considering that long-term knowledge and problem-solving experience may be forgotten, the impact of forgetting on learning and problem-solving skills is modeled by the Ebbinghaus forgetting curve; then, considering the influence of knowledge states of past significant moments on the prediction of current moments, the LSTM layer is designed to aggregate the knowledge states of past significant moments; finally, this paper improves the interpretability of the model by combining traditional IRT. Each parameter in IRT (learning ability, item attributes, etc.) has a specific meaning, so combining IRT can explain the parameter level.

## DKVMN&MRI

This section gives the general framework and each block of the deep knowledge tracing model of DKVMN&MRI, including the input layer, DKVMN layer, LSTM layer, prediction layer, and the training technique of the model.

### General framework of the model

**Fig 2:**
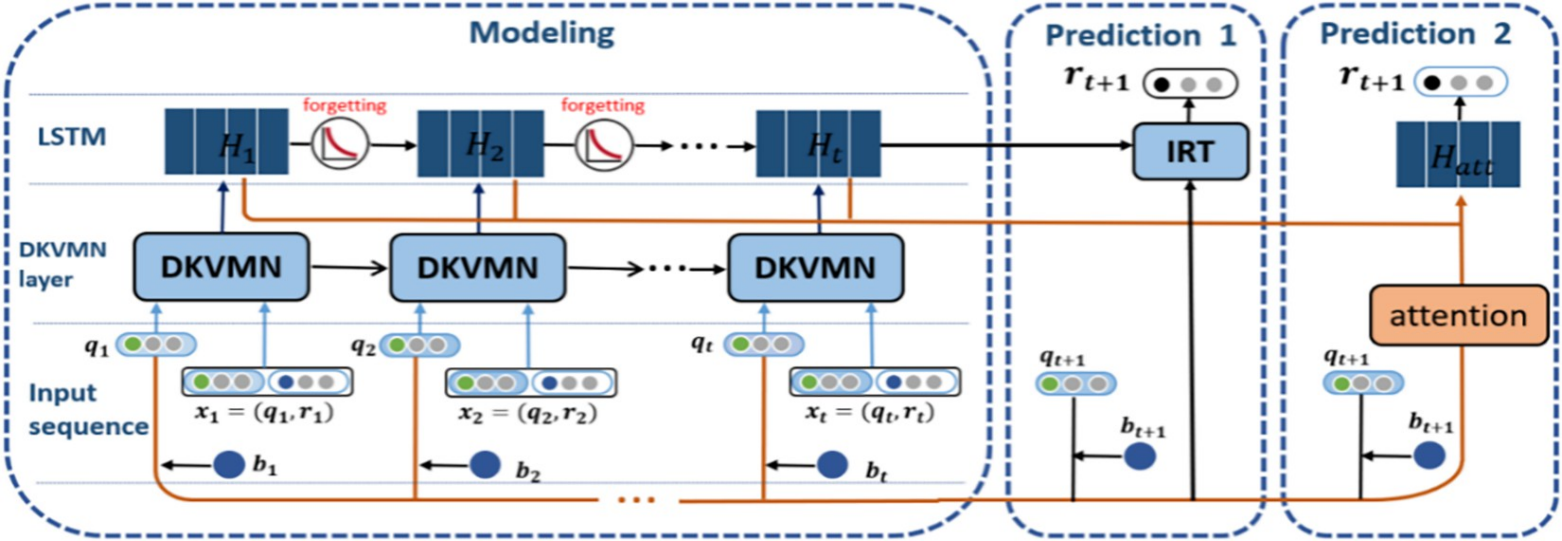
DKVMN&MRI model diagram.

The deep knowledge tracing model of DKVMN&MRI proposed in this paper is primarily split into two components: learning process modeling and answer prediction modeling. As in Fig 2, the input layer, DKVMN layer, and LSTM layer make up the learning process modeling portion. The learner answer prediction part is designed with an IRT-based prediction strategy and an attention mechanism-based prediction strategy.

**Input layer:** The inputs to the model are the student learning log ***x***_***t***_ and the exercise-knowledge point embedding ***q***_***t***_ mapped by the Q matrix.

**DKVMN layer:** DKVMN maps the input Q matrix to a static matrix storing knowledge points and based on the answer scores of the current exercises to dynamically updates the state of the corresponding knowledge points.

**LSTM layer:** The LSTM layer will update the students’ knowledge mastery status corresponding to each moment and introduce the Ebbinghaus forgetting curve function to calculate the degree of influence of experience in doing questions on the current test prediction.

**Prediction layer:** (1) the IRT-based prediction strategy makes the assumption that the exercise’s difficulty and the student’s existing knowledge level characteristics can be used to compute the probability that a student will correctly answer the current exercise; (2) the attention mechanism-based prediction technique makes the assumption that a student’s ability to correctly answer the current exercise depends on their knowledge state at important moments in the past.

### Q-matrix and learning log for knowledge point-test association

Q-matrices are a bridge to achieve the connection between cognition and measurement [27], and researchers map the relevance of knowledge points and exercises to Q-matrices. Usually, Q-matrices are labelled by experts or teachers in the relevant subject, and they have an important role in areas such as cognitive diagnosis. Tatsuoka [28] introduced Q-matrices with 0 or 1 to express the relationship between attributes (knowledge, skills, strategies, etc.) and exercises. By utilizing the Q matrix, the attributes of the test items can be clarified, and cognitive diagnosis can provide the cognitive defects and advantages of the test-takers.

**Fig 3:**
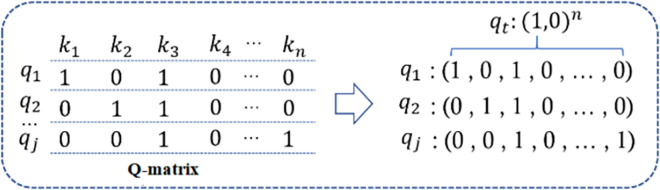
Q-matrix coded exercise vector.

In this paper, the mapping relationship between knowledge points and exercises is modelled as an input to the model using Q-matrices, as shown in Fig 3. Exercise ***q***_**1**_ contains knowledge points ***k***_**1**_ and ***k***_**3**_, and the corresponding position is marked as 1 in the Q-matrix. If the position element is 0, the exercise does not involve the corresponding knowledge point. Then ***q***_**1**_ is encoded as an n-dimensional exercise vector, the values of dimensions 1 and 3 are set to 1, and the values of the remaining dimensions are set to 0. Therefore, the exercise vector can be represented as an n-dimensional vector of 0 and 1.

**Fig 4:**
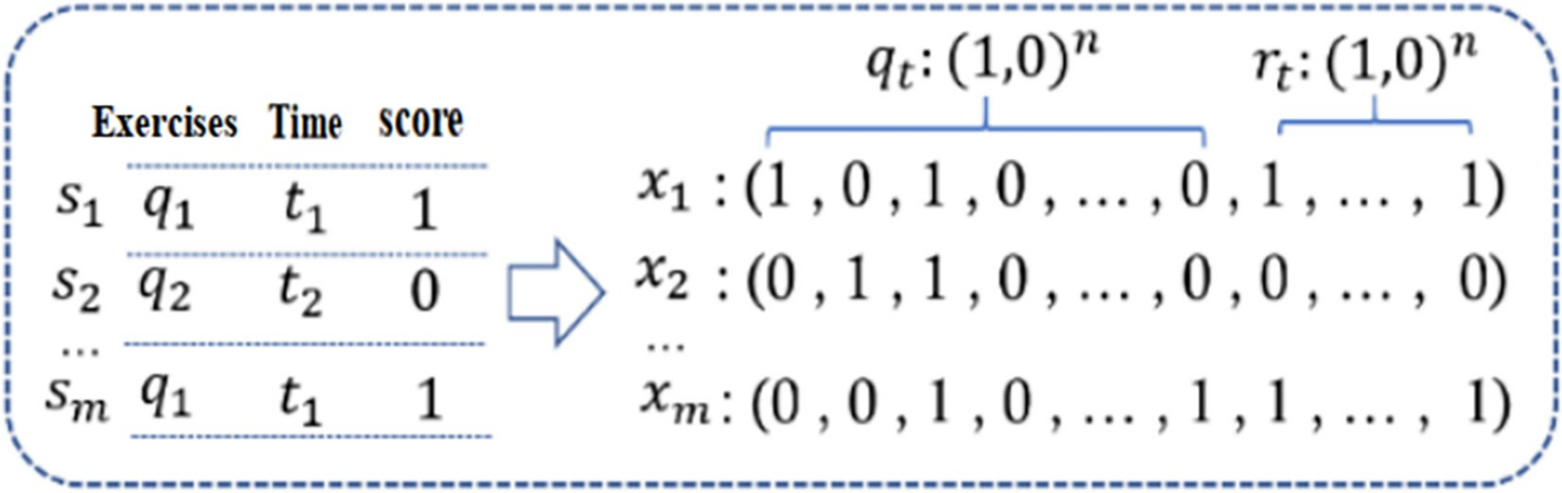
The learning log vector consists of exercises and scores.

In addition to inputting the mapping relationship between knowledge points and exercises into the model, another aspect is encoding the learner’s learning log. As in Fig 4, student ***s***_**1**_ scores 1 for exercise ***q***_**1**_ at moment ***t***_**1**_. This learning log is encoded as a concatenation of the exercise vector with the score vector. Specifically, if the exercise score is 1, ***x***_***t***_ is the practice vector ***q***_***t***_ spliced with an n-dimensional all-1 vector. If the exercise is scored as 0, ***x***_***t***_ is the practice vector ***q***_***t***_ spliced with an n-dimensional all-0 vector, and the process is formulated as follows:

$$x_{t}=\{\begin{array}{c} {{(q}_{t},1)}^{2n}\;if\;r_{t}=1\\ {\left(q_{t},0\right)}^{2n}\;if\;r_{t}=0 \end{array}$$ (1)

### DKVMN layer for modeling the learning process

**Fig 5:**
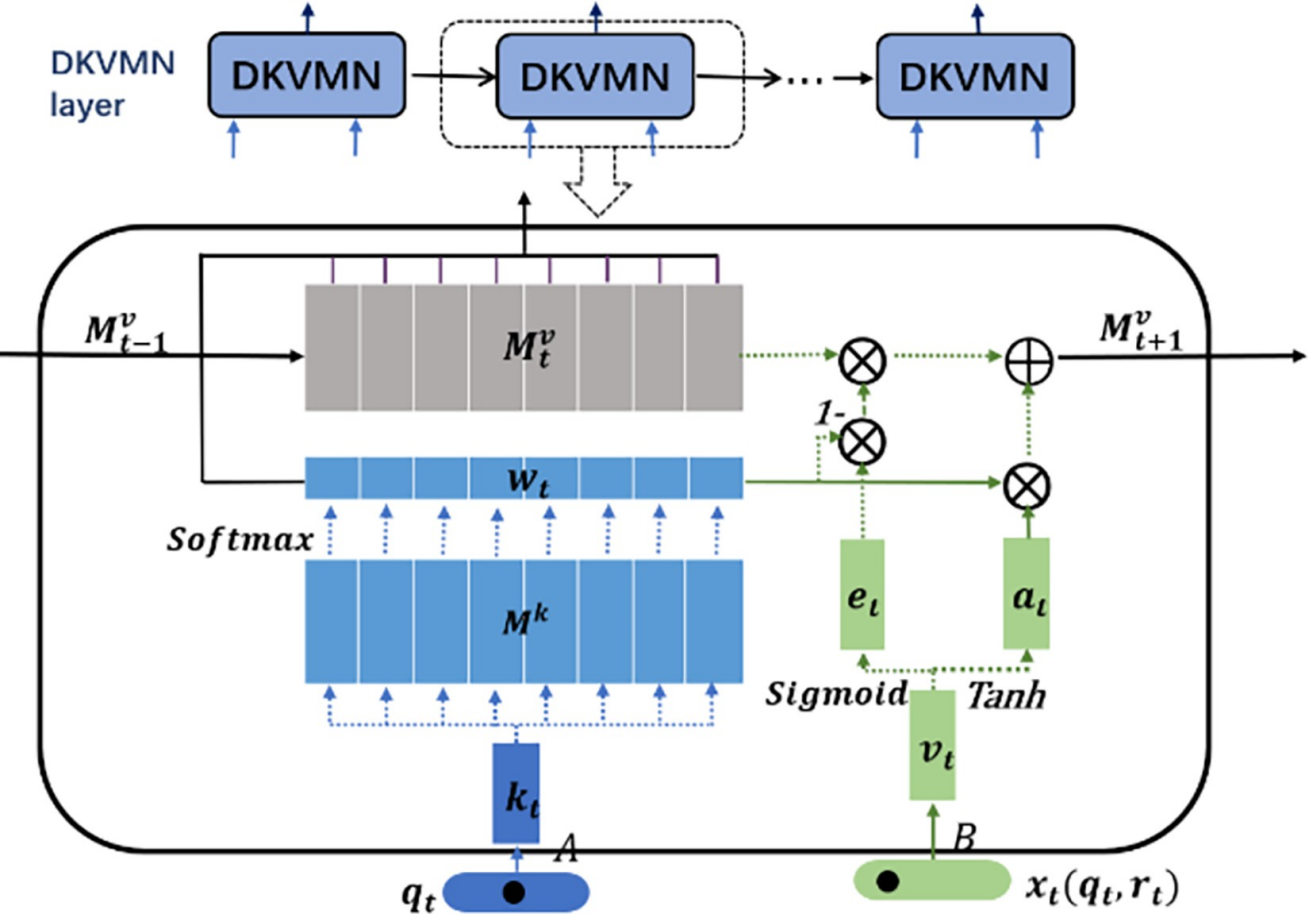
Internal structure of DKVMN.

Inspired by computer architecture, a neural network similar to external memory storage: Memory-Augmented Neural Network (MANN) was proposed by researchers to enhance the network’s capacity to identify long-term dependencies of sequences and allow advances in fields including Question-Answering [29], Natural Language Processing (NLP) [30], Algorithmic Reasoning [31], and One-Shot Learning [32]. Shi et al. further extended a dynamic matrix based on MANN to enhance the ability of MANN to track the mastery status of specific knowledge points. However, there are still limitations in applying DKVMN to the knowledge tracing field:

Firstly, DKVMN maps all knowledge points to a static matrix. When receiving exercise scores from the learning log, it updates the mastery status of the corresponding knowledge points. It reads the mastery status of the knowledge points in predicting a new exercise score. This prediction mechanism treats exercises containing the same knowledge points as identical. However, in reality, the relationships between exercises may be complex in terms of the attributes being tested (e.g., knowledge points, skills, and strategies) and their inherent properties (e.g., difficulty and discriminability). Secondly, when predicting the score of the current exercise, DKVMN directly reads the mastery status of the corresponding knowledge points and does not consider the influence of the knowledge status of important past moments on the prediction of the current moment. The experience of answering similar exercises is an important reference object for predicting the current exercise, so combining the knowledge state of important past moments is necessary. Therefore, this paper does not take the knowledge state of the dynamic matrix in DKVMN as the final state to evaluate students’ knowledge level but as a transition state to input its results to the next layer. The DKVMN layer is described in detail next, and its structure is illustrated in Fig 5.

The inputs to the DKVMN layer are the practice vector ***q***_***t***_ and the learning log ***x***_***t***_ encoded by the Q matrix. Next, ***q***_***t***_ and ***x***_***t***_ are mapped into the ***d***_***k***_-dimensional ***k***_***t***_ and ***v***_***t***_ vectors using the transformation matrices $A\in R^{n\times d_{k}}$ and $B\in R^{2n\times d_{k}}$, respectively, and the weights ***w***_***t***_ are obtained by multiplying ***k***_***t***_ with each column of the knowledge space matrix ***M***^***k***^:

$$w_{t}^{i}=\mathrm{Softmax}\left(k_{t}^{T}M_{i}^{k}\right)=\frac{exp(k_{t}^{T}M_{i}^{k})}{\sum_{i=1}^{K}\left(exp(k_{t}^{T}M_{i}^{k})\right)}$$ (2)

The weight indicates the relevance of ***q***_***t***_ to all knowledge points in the knowledge space, and ***M***^***k***^ stores the embedded representation of all knowledge. The green part of the component shows the process of updating the dynamic matrix $M_{t}^{v}$ after obtaining the current exercise score. Firstly, an erasing vector **e**_**t**_ will be calculated according to ***v***_***t***_. The idea of this process is similar to the forget gate of LSTM, where a part of the information is erased according to the current input, and the amount of information erased is determined by weight ***w***_***t***_. The calculation process is as follows:

$$e_{t}=\mathrm{Sigmoid}(E^{T}v_{t}+b_{e})$$ (3)

$$\bar{M_{t}^{v}}(i)=M_{t-1}^{v}(i)[1-w_{t}(i)e_{t}]$$ (4)

Where **E** is a transformation matrix of ***d***_***k***_×***d***_***k***_. Next, an additive vector ***a***_**t**_ is calculated based on the current ***v***_***t***_. Finally, the knowledge state stored in the dynamic matrix is updated based on ***a***_**t**_. The process is calculated as follows:

$$a_{t}=\mathrm{Tanh}{\left(D^{T}v_{t}+b_{a}\right)}^{T}$$ (5)

$$M_{t}^{v}(i)=\bar{M_{t-1}^{v}}(i)+w_{t}(i)a_{t}$$ (6)

### LSTM layer and learning forgetting mechanism

LSTM is a variant of recurrent neural network with excellent performance for dealing with long sequence dependency problems. Unlike RNN, LSTM controls the updating of the cell state at each time step by setting the input, forget, and output gates. This paper uses the LSTM to update the final knowledge state at each moment, i.e., $H_{t}^{i}=LSTM(M_{t}^{v}(i),H_{t-1}^{i},\theta_{H^{i}})$. The particular procedure for computation is as follows:

$$i_{t}=\sigma (Z_{xi}M_{t}^{v}+Z_{hi}H_{t-1}^{i}+b_{i})$$ (7)

$$f_{t}=\sigma (Z_{xf}M_{t}^{v}+Z_{hf}H_{t-1}^{i}+b_{f})$$ (8)

$$o_{t}=\sigma (Z_{xo}M_{t}^{v}+Z_{ho}H_{t-1}^{i}+b_{o})$$ (9)

$$c_{t}=f_{t}∙c_{t-1}+i_{t}∙\tanh\left(Z_{xc}M_{t}^{v}+Z_{hc}H_{t-1}^{i}+b_{c}\right)$$ (10)

$$H_{t}^{i}=o_{t}∙\tanh\left(c_{t}\right)$$ (11)

Where ***Z***_x*_, ***Z***_h*_, and b_*_ are network parameters. Although the forget gate in the LSTM structure can control the partial discard of information from the current cell state to the next, but it is insufficient for updating the knowledge state. Studies on memory forgetting by psychologist Ebbinghaus [33] have shown that human memory decays over time according to the Ebbinghaus forgetting curve function. Therefore, it is necessary to consider forgetting factors when modelling learners’ knowledge states, and the specific procedure will be explained in section of “Prediction Strategy Based on Attention Mechanism”.

### Prediction strategy based on IRT-Rasch model

Item Response Theory (IRT) is a classic psychometrics theory that uses a mathematical model containing unknown parameters to reveal the relationship between test-takers’ ability and answer accuracy [34]. Among them, the Danish psychologist Rasch initially introduced the one-parameter Rasch model [35], whose expression is as follows:

$$P(y_{ij}=1|\theta_{i},b_{j})=\frac{1}{1+e^{-(\theta_{i}-b_{j})}}$$ (12)

Where **P** represents the probability that test-taker **i** answers item **j** correctly, ***θ***_***i***_ describes the potential traits of test-taker **i** (such as ability and knowledge state), and ***b***_***j***_ describes the difficulty of item **j**. Among them, **θ** is closely related to the knowledge mastery level of the test-taker. If the test-taker grasps a knowledge point well, its corresponding parameter **θ** is larger. **θ** can be described by the corresponding knowledge state of the test-taker:

$$v_{t}=q_{t}C$$ (13)

$$\theta_{i}=\mathrm{Sigmoid}(\sum_{j=1}^{n}{(v}_{t}^{T}H_{t}^{j}))$$ (14)

Where C is the transition matrix of, and is column i in the knowledge state.

In the IRT, the difficulty parameter ***b***_***j***_ describes the ability point with a probability of 0.5 for correctly answering the item. The potential of properly answering a question of the same difficulty increases when the ability parameter of the test-taker is large. Similarly, subjects of the same ability were more likely to answer the less difficult questions correctly. In this article, ***b***_***j***_ is defined as:

$$b_{j}=\frac{\sum_{i=1}^{s}C_{q_{j({i=j,r}_{i}=1)}}}{\sum_{i=1}^{s}C_{q_{j(i=j)}}}$$ (15)

$\sum_{i=1}^{s}C_{q_{j({i=j,r}_{i}=1)}}$ represents the number of times that exercise **j** has been answered correctly in the question bank, and $\sum_{i=1}^{s}C_{q_{j(i=j)}}$ represents the number of times that exercise **j** has appeared. Finally, by combining the Rasch model of IRT, the possibility that student i would correctly answer exercise j is predicted:

$$P(y_{i,j=1}|\theta_{i},\;b_{j})=\frac{1}{1+e^{-D(\theta_{i}-b_{j})}}$$ (16)

Where, **D** is a constant, typically taking the value of 1 or 1.7. When D = 1, it indicates the use of a logistic scale, while D = 1.7 indicates the use of a normal ogive metric. When D = 1.7, Eq (16) closely approximates the standard normal distribution curve [36].

### Prediction strategy based on attention mechanism

Predictive strategies based on attention mechanism can focus on the state of knowledge at important moments in the past. Specifically, when a student faces an exercise similar to the one in the past practice record, the results of the answer at that time may have a high correlation with the predicted results. This correlation increases with the increase of the similarity between the exercises. The similarity between exercises is not only related to the knowledge they involve but also to the difficulty. Even if two exercises containing the same knowledge point are pretty different in difficulty, their similarity will be low. Therefore, this paper describes the similarity of the two exercises from the knowledge level and the difficulty level:

$$\alpha_{ij}=cos(q_{i},q_{j})$$ (17)

$$\beta_{ij}=\lambda \alpha_{ij}+(1-\lambda )(1-|b_{i}-b_{j}|)\;(\lambda \in (0,1)$$ (18)

Where, ***α***_***ij***_ is the cosine similarity of ***q***_***i***_ and ***q***_***j***_, which describes the similarity of exercise **i** and exercise **j** in knowledge level. ***λ*** is an adjustable parameter, ***b***_***i***_ and ***b***_***j***_ are the difficulty of exercise **i** and exercise **j**, respectively, where a smaller difference indicates that they are more similar at the difficulty level. Then, the similarity reflected by the two levels is fused to obtain the similarity ***β***_***ij***_ of exercise **i** and exercise **j**.

However, it is not enough to describe the knowledge state of essential moments in the past only through the level of knowledge and exercise difficulty. Human learning is also accompanied by the forgetting effect. Specifically, when learners are faced with a new exercise, they search for relevant knowledge or experience in their brain, which is formed in the previous learning or experience but may be forgotten as time passes. For learners, knowledge or experience learned over a long period may have less influence on current problem-solving. This paper quantifies the influence of forgetting factors on prediction by introducing the Ebbinghaus forgetting curve function:

$$\Delta t={|t_{i}-t}_{j}|$$ (19)

$${\tilde{\beta }}_{ij}=exp{(-\mu ∙\Delta t)∙\beta }_{ij}\;(\mu >0)$$ (20)

**Fig 6:**
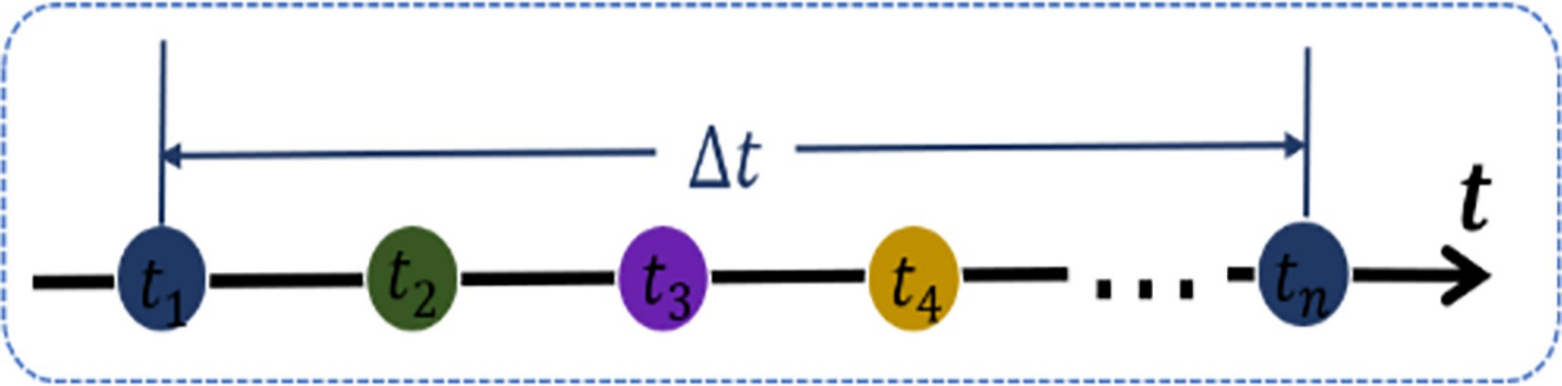
Forgetting effect described.

Where, ***μ*** is the parameter that is always greater than 0, ${\tilde{\beta }}_{ij}$ is the similarity of exercise **i** and exercise **j** depicted at the forgetting level, and Δ***t*** is the measure of two-time steps. As shown in Fig 6, for learners, exercises at moment ***t***_**1**_ with the same color are similar to exercises at the current moment, but this similarity gradually decreases over time. This method of quantifying the decay of past problem-solving experience over different time steps characterizes the impact of forgetting on learning.

Thus, the similarity between exercise **i** and exercises at each past moment can be expressed as ${\tilde{\beta }}_{*i}$ = (${\tilde{\beta }}_{1t}$, ${\tilde{\beta }}_{2t}$, ${\tilde{\beta }}_{3t}$,…,${\tilde{\beta }}_{i-1,i},0,\ldots ,0$) vector of dimension **t**, ${\tilde{\beta }}_{ji}=0$ when j ≥ i. Then, by aggregating the knowledge state of important moments in the past, the calculation process is as follows:

$$H_{att}^{i}=\sum_{j=1}^{t}{\tilde{\beta }}_{*i}H_{j}^{i}$$ (21)

$$y_{t+1}=ReLU(W_{1}∙[\sum_{i=1}^{K}{\tilde{\beta }}_{*i}H_{att}^{i}⨁q_{t+1}]+b_{1})$$ (22)

$$r_{t+1}=Sigmoid(W_{2}∙y_{t+1}+b_{2})$$ (23)

Where Formula (21) is the polymerization process of knowledge state, ***y***_***t*+1**_ is the representation of prediction information, ***W***_**1**_、***W***_**2**_、***b***_**1**_ and ***b***_**2**_ are the model parameter, and ***r***_***t*+1**_ represents the students’ performance of prediction in ***q***_***t*+1**_. By further setting a threshold, if the value of ***r***_***t*+1**_ is greater than this threshold, it means that the student can answer ***q***_***t*+1**_ correctly.

### Objective functions

The negative log-likelihood function based on students’ learning sequence scores is this model’s objective function:

$$L=-\sum_{t=1}^{T}\left({\overset{ˇ}{r}}_{t}logr_{t}+(1-{\overset{ˇ}{r}}_{t})\log\left(1-r_{t}\right)\right)$$ (24)

Where ${\overset{ˇ}{r}}_{t}$ represents the score of the ground truth label, and ***r***_***t***_ for the predicted label’s score. The parameters in the model are updated by minimizing the predicted probability and the crossentropy loss of the true label results using stochastic gradient descent.

## Evaluation

This section first introduces the dataset, comparison models, and evaluation metrics used in the experiment. Subsequently, it presents the experimental results and analyzes them, demonstrating the superiority of the proposed model and explaining the reasons for performance variations across different datasets. Ablation experiments are conducted to validate the necessity and effectiveness of each component of the model. Then, visual tracking of student knowledge states confirms the model’s interpretability and its capability to model learner forgetting behaviors. Finally, by analyzing dataset difficulty and clustering of similar exercises using the model, it is demonstrated that the model effectively captures relationships between exercises.

### Data sets, comparison models and evaluation methods

Experiments were carried out using actual data gathered from three online learning platforms in order to assess the efficacy of the suggested approach in this study. The description of the dataset is provided below:

1. **ASSIST2009:** ASSISTments online education platform generated this dataset, which has been used in several articles to assess knowledge tracing models. After deduplication, it contains 325,673 interaction records from 4151 students across 110 questions.
2. **ASSIST2015:** This dataset includes 708,631 entries from 19,917 students covering 100 knowledge points. The greater number of students results in a lower average number of interactions per student even if there are more records than in ASSIST2009.
3. **ASSISTchall:** The 2017 ASSISTments Data Mining Competition made use of this dataset. On average, there are more records per student, with a total of 942,816 interactions collected from 686 students across 102 knowledge points.
4. **EdNet:** The dataset is contributed by Choi et al. in [37]. It is a large dataset with over 130 million records that involve about 0.78 million students. We sampled 225,625 interaction records of 5000 students at 188 knowledge points from the dataset.

To confirm that the model presented in this study is effective, seven typical baseline models were selected for comparison in the experiments, including:

1. **BKT:** By modeling the learning process as a hidden Markov model, the mastery of knowledge is represented as a binary variable of mastered or unmastered. This approach views the learning process as a discrete transition from an unmastered state to a mastered state for each knowledge point.
2. **DKT:** Introducing deep learning into the field of knowledge tracing, we use recurrent neural networks to model students’ knowledge states at each input time step.
3. **DKVMN:** By adopting a storage matrix approach to model the mastery of each knowledge point by the student, this method addresses the issue of insufficient handling of long sequence dependencies in DKT.
4. **GIKT** [38]: Using Graph Convolutional Networks (GCN) to model the question-knowledge point correlations, GIKT summarizes the student’s mastery of knowledge points as the student’s present state, the student’s historical related exercises, the goal question, and the interaction between relevant knowledge points.
5. **EERNN:** Taking into account the semantic information of exercises, and using the semantic representation of exercises as model input, EERNN considers that exercises with the same knowledge point may lead to biased prediction results due to semantic differences.
6. **LPKT** [39]: Giving equal attention to correct and incorrect answers, LPKT assumes that students can still learn from incorrect answers.
7. **ATKT** [40]: Using adversarial training to enhance the generalization ability of knowledge tracing, ATKT proposes an aggregation module of knowledge hidden states, emphasizing the importance of the current knowledge state.
8. **ENKT** [41]: Using ensemble learning to model the interactions among students, concepts and exercises during the learning process.
9. **GFLDKT** [42]: two gating-controlled mechanisms are designed to model explicitly forgetting and learning behaviors in students’ learning process. With the designed gating-controlled mechanisms, both the interaction records and students’ different backgrounds are combined effectively for tracing the dynamic changes of students’ mastery of knowledge concepts.

Following the research work of most knowledge tracing models, this experiment adopted accuracy (ACC) and area under the receiver operating characteristic curve (AUC) as evaluation metrics from a classification perspective. AUC evaluates the probability that a positive example is ranked higher than a negative example in the prediction. The formulas for calculating these metrics are as follows:

$$\mathrm{ACC}=\frac{r}{N}$$ (25)

$$\mathrm{AUC}=\frac{\sum_{i\in positive}{rank}_{i}-\frac{m(1+m)}{2}}{m\times n}$$ (26)

Where **N** represents the total number of exercise interactions for students, **r** represents the number of correct predictions, **m** represents the number of positive examples, and **n** represents the number of negative examples.

### Performance comparison

Each dataset has 80% of its data divided into training and validation sets, with the remaining 20% serving as the test set. Table 1 presents the results of the experiment, where the best performance achieved for each metric on the corresponding dataset is highlighted in bold black text.

**Table 1:** Experimental results and performance comparison.

Model	ASSIST 2009		ASSIST 2015		ASSIST chall		EdNet	
	ACC	AUC	ACC	AUC	ACC	AUC	ACC	AUC
BKT	0.611	0.648	0.601	0.616	0.559	0.562	0.570	0.591
DKT	0.721	0.754	0.715	0.723	0.714	0.729	0.692	0.718
DKVMN	0.702	0.710	0.701	0.705	0.698	0.712	0.696	0.712
GIKT	0.742	0.778	0.735	0.773	0.716	0.736	0.712	0.735
EERNN	0.748	0.765	0.756	0.793	0.731	0.769	0.725	0.757
LPKT	0.752	0.778	0.769	0.805	0.743	0.802	0.738	0.768
ATKT	0.779	0.820	0.766	0.804	0.763	0.792	0.756	0.774
ENKT	0.774	0.810	0.764	0.795	0.760	0.784	0.745	0.760
GFLDKT	0.778	0.821	0.767	0.804	**0.771**	0.804	0.752	0.771
DKVMN&MRI-IRT	0.775	0.812	0.768	0.797	0.761	0.798	0.762	0.789
DKVMN&MRI-ATT	**0.785**	**0.828**	**0.771**	**0.817**	0.770	**0.806**	**0.765**	**0.792**

Experimental results indicate that the BKT model, representing conventional methods, performs poorly across all datasets and exhibits significant gaps compared to other deep knowledge tracing models. In contrast to traditional machine learning-based models, DKT and DKVMN demonstrate superior performance, highlighting the powerful representation learning capabilities of deep learning models. However, the performance of these two models still lags behind other deep learning-based KT models. This is primarily because these models only consider knowledge point IDs and learner response data, with limited input information. GIKT utilizes graph convolutional networks to extract relationships between exercises and knowledge points. EERNN incorporates semantic information from exercises. LPKT focuses on learning information at moments of incorrect responses. ENKT considers interactions among students, concepts, and exercises during the learning process. GFLDKT integrates information about the relationship between student learning and forgetting. The incorporation of these relation information enables the knowledge tracing model to have better predictive ability. ATKT enhances the generalization ability of knowledge tracing through adversarial training, resulting in superior model performance. However, these models do not fully consider the relational information necessary for precise knowledge tracing of students. The proposed DKVMN&MRI model captures the relationships between exercises and knowledge points by introducing a Q-matrix. It then models the learner’s learning process using LSTM and an improved DKVMN, and simulates the learner’s memory forgetting process using the Ebbinghaus forgetting curve function. This approach effectively captures the relationship between students’ procedural interaction data and behaviors such as knowledge acquisition and forgetting. Compared to the modeling methods of various baseline models, this represents a more efficient and interpretable approach to human cognitive modeling. Finally, two prediction strategies were employed, combining the learner’s knowledge state with the similarity between exercises to calculate the probability of correctly answering the next question at the subsequent time step. We conducted comparative experiments on four datasets: ASSIST2009, ASSIST2015, ASSISTchall and EdNet. We computed and recorded the evaluation metrics, ACC and AUC, for the DKVMN&MRI model as well as other baseline models. The experimental results indicate that the proposed model significantly outperforms the other baseline models. Among the methods proposed, DKVMN&MRI-IRT and DKVMN&MRI-ATT are based on IRT and attention mechanisms, respectively, with the attention-based method achieving the best results in both metrics.

Furthermore, the four datasets differ in several aspects, such as the number of questions, difficulty levels, and the number of knowledge points, which can affect the model’s performance across these datasets. In the experiments, most models performed well on the ASSIST2009 dataset, which has a moderate average length of student response sequences, but exhibited poorer performance on the EdNet dataset, which has relatively longer average response sequences. It can be inferred that the length of response sequences is also a significant factor influencing model prediction performance. The proposed model can more accurately capture learning information in datasets with longer student interaction sequences and larger volumes of data, leading to improved predictive outcomes.

### Ablation study

In this section, we conducted ablation experiments on the DKVMN&MRI-ATT model by systematically removing specific modules to gain a detailed understanding of each component’s contribution to the overall model performance. The accuracy results are presented in Table 2. DKVMN&MRI-Q denotes that the Q-matrix is not utilized within the item embedding module. DKVMN&MRI-U indicates the exclusion of considerations regarding forgetting factors in knowledge state updates by removing the LSTM layer, and it utilizes the knowledge state from the dynamic matrix in DKVMN as the final assessment of student knowledge proficiency. DKVMN&MRI-N represents a prediction mechanism that operates without any forecasting strategy, disregarding inter-question relationships, and making predictions solely based on the student’s current knowledge state and the current exercise.

**Table 2:** DKVMN&MRI-ATT model ablation experiment.

Model	ASSIST 2009		ASSIST 2015		ASSIST chall		EdNet	
	ACC	AUC	ACC	AUC	ACC	AUC	ACC	AUC
DKVMN&MRI-Q	0.768	0.793	0.762	0.806	0.763	0.785	0.752	0.784
DKVMN&MRI-U	0.773	0.808	0.768	0.805	0.769	0.793	0.758	0.791
DKVMN&MRI-N	0.762	0.789	0.757	0.791	0.757	0.782	0.746	0.770
DKVMN&MRI-ATT	**0.785**	**0.828**	**0.771**	**0.817**	**0.770**	**0.806**	**0.765**	**0.792**

From the experimental results, it is evident that removing the Q-matrix, LSTM layer, and attention prediction layer all led to varying degrees of performance decline in the model, indicating their positive contributions to predictive capability. Specifically, for DKVMN&MRI-Q, the inability to fully leverage the relationships between questions and knowledge points resulted in decreased predictive performance. For DKVMN&MRI-U, ignoring the learner’s forgetting behavior resulted in decreased predictive performance of the model, highlighting the significant role that simulating forgetting behavior plays in enhancing predictive capability. Regarding DKVMN&MRI-N, its performance exhibited the largest disparity compared to DKVMN&MRI-ATT, indicating that incorporating relational information between questions into the model has the greatest impact on performance improvement.

### Analysis of student knowledge state

**Fig 7:**
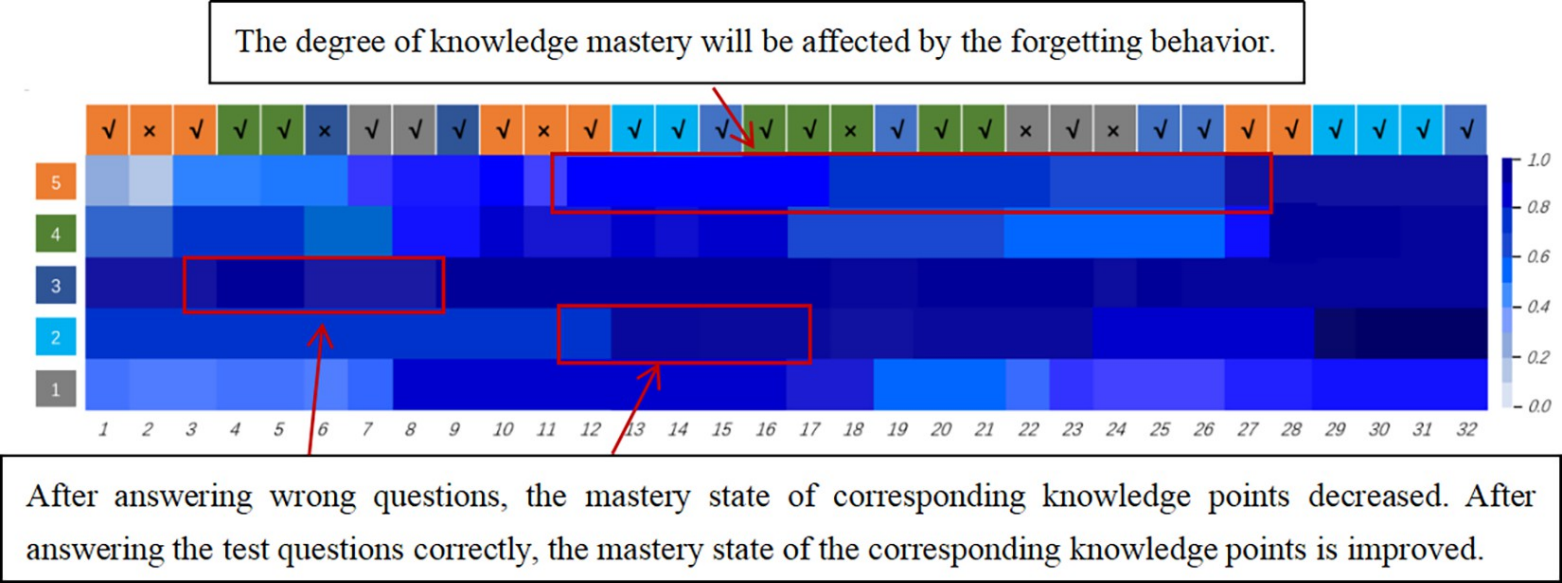
Visualization of knowledge.

While our model achieved good performance, the interpretability of the model is also a crucial aspect for the evaluation of knowledge tracing. To demonstrate the validity of our model in tracing student knowledge states, we visualized the mastery status of a single student across five knowledge components, as shown in Fig 7. The darker color indicates a better mastery status for the corresponding knowledge component, with the left index representing different knowledge components and the top index representing the corresponding exercises. It can be observed that the mastery status of the third knowledge component declined following an incorrect answer to a question at the sixth time step, while the corresponding knowledge component’s mastery status increased following two consecutive correct answers at the 13th and 14th time steps. Therefore, the visualization results demonstrate the validity of the knowledge tracing process.

For the mastery state of the 5th knowledge point, the DKVMN&MRI-IRT model indicates a continuous decline in learner mastery of knowledge point 5 from the 12th time step, where the learner initially learns knowledge point 5, to the 27th time step when revisiting knowledge point 5. This is because as the time interval increases, learners are influenced by forgetting behaviors, leading to a decline in mastery of various knowledge points. Therefore, the visualization results also demonstrate that DKVMN&MRI-IRT effectively simulates learner forgetting behaviors.

### Analysis of inter-exercises relationship information

**Fig 8:**
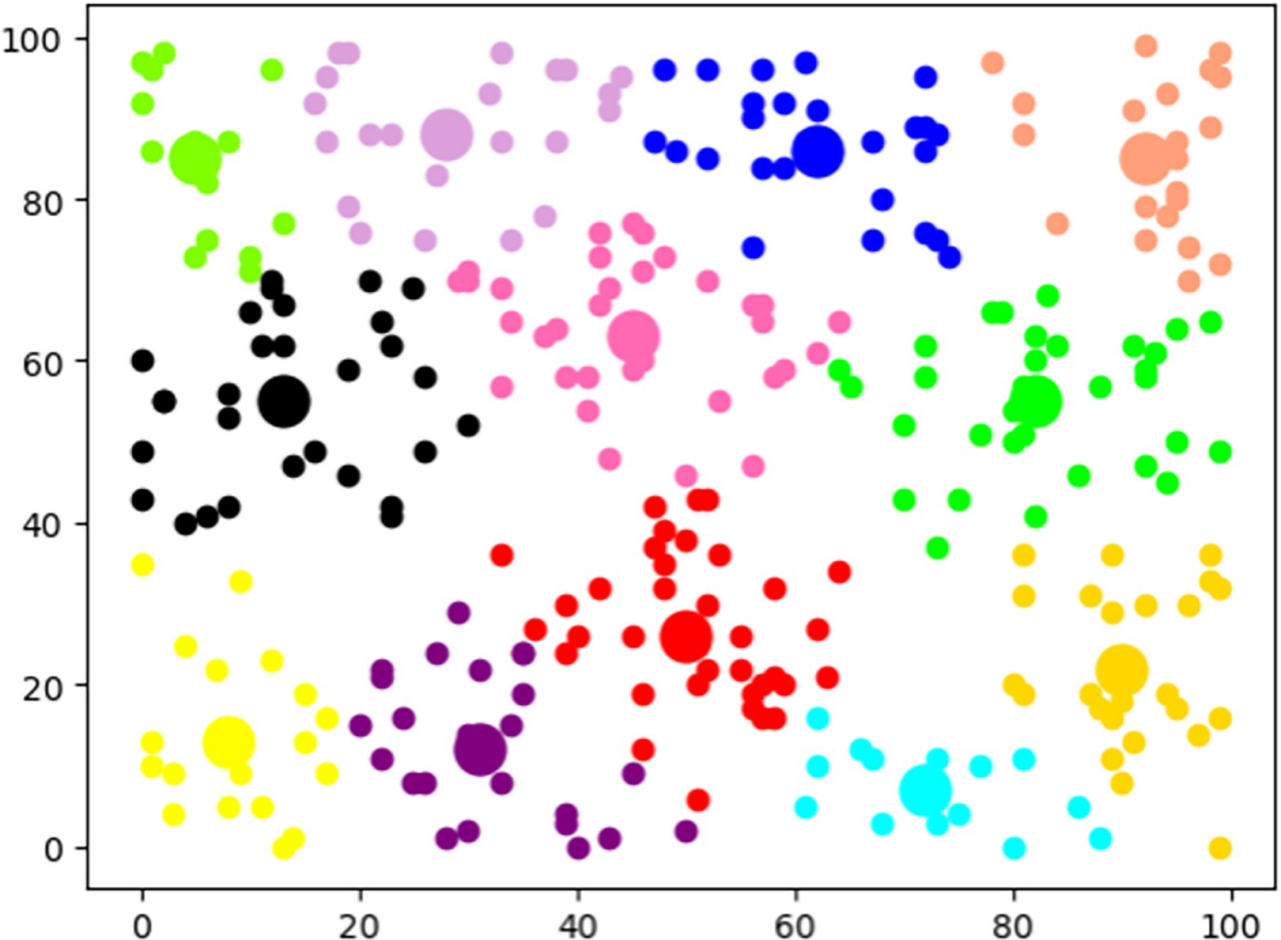
Similarity analysis.

**Fig 9:**
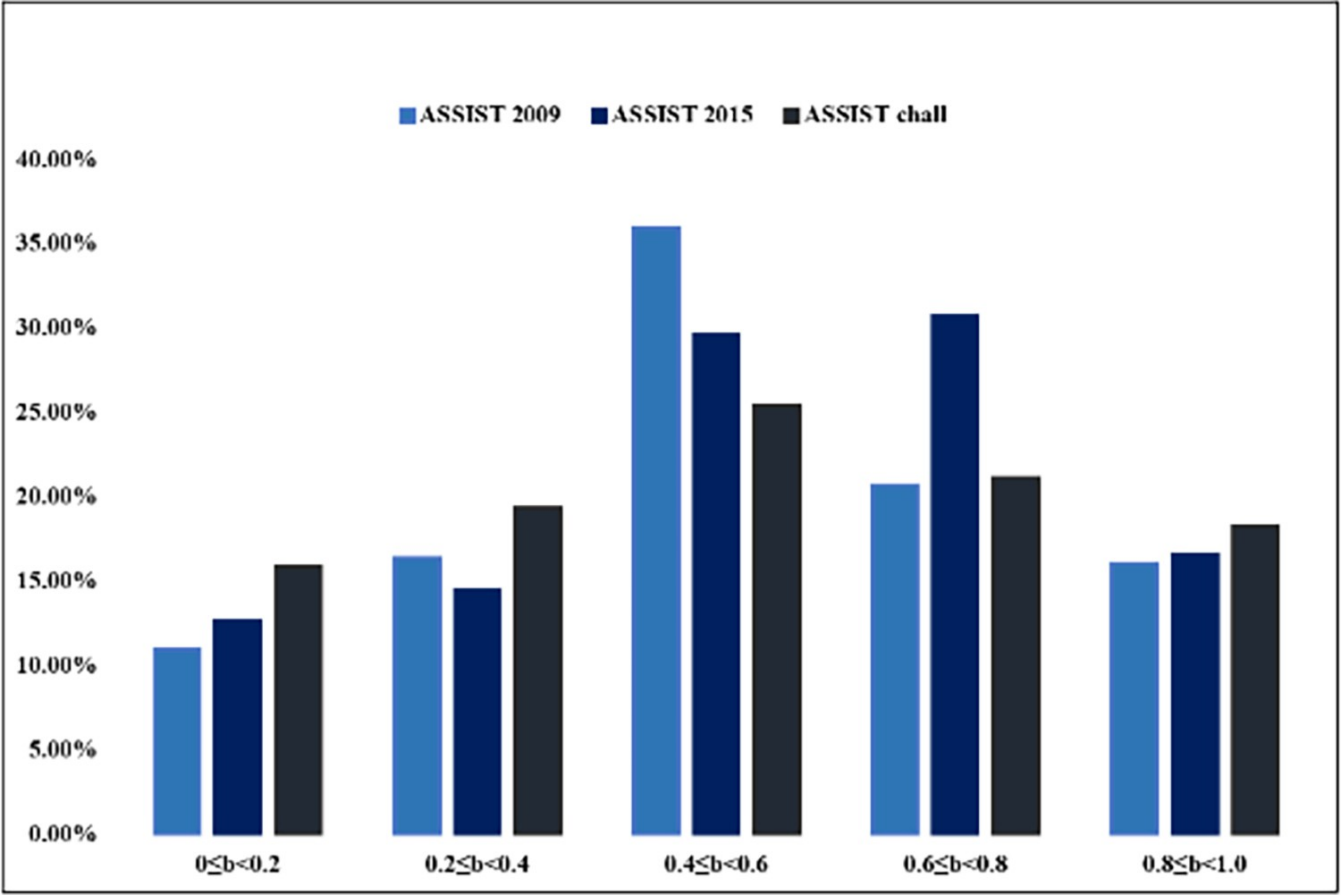
Difficulty analysis of test.

DKVMN&MRI-ATT can capture the interrelatedness between exercises based on their knowledge points and difficulty levels, by calculating the distance between exercise embeddings in space. As shown in Fig 8, we performed clustering analysis on 200 exercises from the ASSIST2009 dataset in the knowledge space. It can be observed that these exercises are classified into 12 categories, with exercises in the same category possibly having the same knowledge point, and exercises closer in distance indicating similar difficulty levels. The results of these automated learning can be used to supplement data in the field of education. In addition, this study also analyzed the difficulty of exercises in three datasets. As shown in Fig 9, it can be observed that the difficulty coefficient of most exercises is distributed between 0.4 and 0.8. Compared to the other two datasets, the exercises in ASSIST-chall are more evenly distributed across each difficulty interval.

## Conclusion and future work

This paper proposes a dynamic key-value memory network model that incorporates the relationships between exercises and knowledge components, exercises and exercises, and learning and forgetting to model knowledge tracing for learners. Firstly, the Q-matrix is introduced to integrate the interaction between knowledge points and exercises into the model. The similarity between exercises is characterized at the knowledge component level and the exercise difficulty level to enable the traditional DKVMN to consider the relationship between exercises. Then, the Ebbinghaus forgetting curve function is incorporated to model the impact of forgetting on learning. Finally, through experiments on three real-world datasets, the proposed model using prediction strategies based on IRT and attention mechanisms, is validated for tracking student knowledge states. The proposed model’s efficacy and interpretability are demonstrated by the experimental results.

In the future, we can introduce the hierarchical relationships between knowledge points as a modeling constraint. Knowledge points are the basic units for transmitting teaching information in the teaching process, including theories, principles, concepts, definitions, examples, and conclusions [43]. These basic knowledge units are not independent of each other but are interrelated and mutually influential, with a certain hierarchical relationship. Relationships, as a part of mathematical models, are usually implicitly expressed in data structures (such as arrays, tables, trees, graphs, etc.) [44]. Introducing the inherent hierarchical relationships between knowledge points in the model can serve as a constraint for knowledge tracing tasks.
